# Complete Genome Sequence of *Parascardovia denticolens* JCM 12538^T^, Isolated from Human Dental Caries

**DOI:** 10.1128/genomeA.00485-15

**Published:** 2015-05-14

**Authors:** Kenshiro Oshima, Jun-ichiro Hayashi, Hidehiro Toh, Akiyo Nakano, Chie Shindo, Keiko Komiya, Hidetoshi Morita, Kenya Honda, Masahira Hattori

**Affiliations:** a Graduate School of Frontier Sciences, The University of Tokyo, Kashiwa, Chiba, Japan; b CREST, Japan Science and Technology Agency, Kawaguchi, Saitama, Japan; c School of Dentistry, Aichi Gakuin University, Nagoya, Japan; d Medical Institute of Bioregulation, Kyushu University, Fukuoka, Japan; e Department of Microbiology and Infectious Diseases, Nara Medical University, Kashihara, Nara, Japan; f Graduate School of Environmental and Life Science, Okayama University, Okayama, Japan; g Department of Microbiology and Immunology, Keio University School of Medicine, Shinjuku-ku, Tokyo, Japan

## Abstract

*Parascardovia denticolens* JCM 12538^T^ was isolated from human dental caries. Here, we report the complete genome sequence of this organism. This paper is the first report demonstrating the completely sequenced and assembled genome of *P. denticolens*.

## GENOME ANNOUNCEMENT

*Parascardovia denticolens* JCM 12538^T^ (=DSM 10105^T^) was originally isolated as *Bifidobacterium denticolens* from human dental caries (1) and then was renamed *P. denticolens* (2). *P. denticolens* is classified under the family *Bifidobacteriaceae*. This species is isolated from human dental caries and plaque (3, 4) and is also found in microbiota associated with peri-implantitis (5).

We determined the complete genome sequence of *P. denticolens* JCM 12538^T^ using the whole-genome shotgun strategy using Sanger sequencing (ABI 3730xl sequencers). We constructed small-insert (2-kb) and large-insert (10-kb) genomic DNA libraries and generated 26,880 sequence reads (10.6-fold coverage) for *P. denticolens* JCM 12538^T^ from both ends of the genomic clones. Data were assembled with the Phred-Phrap-Consed program. Gap closing and resequencing of low-quality regions were conducted by Sanger sequencing to obtain the high-quality finished sequence. The overall accuracy of the finished sequence was estimated to have an error rate of <1 per 10,000 bases (Phrap score of ≥40). An initial set of predicted protein-coding genes was identified using Glimmer 3.0 (6). Genes consisting of <120 base pairs (bp) and those containing overlaps were eliminated. The tRNA genes were predicted by the tRNAscan-SE (7), and the rRNA genes were detected by BLASTn search using known *Parascardovia* rRNA sequences as queries.

The genome sequence of *P. denticolens* JCM 12538^T^ consists of a circular chromosome of 1,890,857 bp with no plasmid. The genome size is smaller than those of the *Bifidobacterium* species, whose genomes range in size from 1.9 to 2.8 Mbp (8). JCM 12538^T^ contained a clustered regularly interspaced short palindromic repeats (CRISPR) (9) region (1,334,669 to 1,340,788), and seven CRISPR-associated genes (PSDT_1094 to PSDT_1100) were encoded upstream of the CRISPR region. The chromosome contains 1,548 predicted protein-coding genes.

*P. denticolens* JCM 12538^T^ is related to *Scardovia inopinata* in the phylogenetic tree of the family *Bifidobacteriaceae* (10). Then, we compared the genome of JCM 12538^T^ with that (1,797,862 bp) of *S. inopinata* JCM 12537^T^ (GenBank accession no. AP012334). Of the 1,548 protein-coding genes, 1,070 (69%) were conserved in the two strains. The genome information of this species will be useful for further studies of its physiology, taxonomy, clinical aspects, and ecology.

### Nucleotide sequence accession number.

The sequence data for the genome have been deposited in DDBJ/GenBank/EMBL under the accession no. AP012333.

## References

[1] Crociani F, Biavati B, Alessandrini A, Chiarini C, Scardovi V 1996 *Bifidobacterium inopinatum* sp. nov. and *Bifidobacterium denticolens* sp. nov., two new species isolated from human dental caries. Int J Syst Bacteriol 46:564–571. doi:10.1099/00207713-46-2-564. 8934909

[2] Jian W, Dong X 2002 Transfer of *Bifidobacterium inopinatum* and *Bifidobacterium denticolens* to *Scardovia inopinata* gen. nov., comb. nov., and *Parascardovia denticolens* gen. nov., comb. nov., respectively. Int J Syst Evol Microbiol 52:809–812. doi:10.1099/ijs.0.02054-0. 12054242

[3] Modesto M, Biavati B, Mattarelli P 2006 Occurrence of the family *Bifidobacteriaceae* in human dental caries and plaque. Caries Res 40:271–276. doi:10.1159/000092237. 16707878

[4] Mantzourani M, Fenlon M, Beighton D 2009 Association between *Bifidobacteriaceae* and the clinical severity of root caries lesions. Oral Microbiol Immunol 24:32–37. doi:10.1111/j.1399-302X.2008.00470.x. 19121067

[5] Tamura N, Ochi M, Miyakawa H, Nakazawa F 2013 Analysis of bacterial flora associated with peri-implantitis using obligate anaerobic culture technique and 16S rDNA gene sequence. Int J Oral Maxillofac Implants 28:1521–1529. doi:10.11607/jomi.2570. 24278920

[6] Delcher AL, Bratke KA, Powers EC, Salzberg SL 2007 Identifying bacterial genes and endosymbiont DNA with Glimmer. Bioinformatics 23:673–679. doi:10.1093/bioinformatics/btm009. 17237039 PMC2387122

[7] Schattner P, Brooks AN, Lowe TM 2005 The tRNAscan-SE, snoscan and snoGPS Web servers for the detection of tRNAs and snoRNAs. Nucleic Acids Res 33:W686–W689. doi:10.1093/nar/gki366. 15980563 PMC1160127

[8] Bottacini F, Ventura M, van Sinderen D, O’Connell Motherway M 2014 Diversity, ecology and intestinal function of bifidobacteria. Microb Cell Fact 13:S4. doi:10.1186/1475-2859-13-S1-S4. 25186128 PMC4155821

[9] Horvath P, Coûté-Monvoisin AC, Romero DA, Boyaval P, Fremaux C, Barrangou R 2009 Comparative analysis of CRISPR loci in lactic acid bacteria genomes. Int J Food Microbiol 131:62–70. doi:10.1016/j.ijfoodmicro.2008.05.030. 18635282

[10] Felis GE, Dellaglio F 2007 Taxonomy of lactobacilli and bifidobacteria. Curr Issues Intest Microbiol 8:44–61. 17542335

